# The neuroelectrophysiological and behavioral effects of transcranial direct current stimulation on executive vigilance under a continuous monotonous condition

**DOI:** 10.3389/fnins.2022.910457

**Published:** 2022-09-07

**Authors:** Jing Dai, Hang Wang, Lin Yang, Chunchen Wang, Shan Cheng, Taihui Zhang, Jin Ma, Zhihong Wen, Xinsheng Cao, Wendong Hu

**Affiliations:** School of Aerospace Medicine, Air Force Medical University, Xi’an, China

**Keywords:** vigilance, cognitive function, cognitive fatigue, inhibitory control, event-related potential, brain stimulation

## Abstract

A prolonged period of vigilance task will lead to vigilance decrement and a drop in cognitive efficiency. Although transcranial direct current stimulation (tDCS) can be used to improve cognitive performance following vigilance decrement, the findings in this area of study are inconsistent. This study aims to identify the neuroelectrophysiological and behavioral effects of tDCS over the left dorsolateral prefrontal cortex (DLPFC) on executive vigilance under a continuous monotonous condition. We recruited 29 participants who randomly received 30 min active or sham tDCS before the vigilance task (anode electrode at the left DLPFC, cathode electrode at the right supraorbital area). Participants completed four sessions of vigilance task and five sessions of self-report sleepiness, Oddball task, and Go/Nogo task, for a total of about 5 h. EEG was acquired in real-time throughout the experiment. Repeated measures of ANOVA were utilized to analyze the evolution of each metric with task-on-time. The results demonstrated that subjective arousal state, vigilance performance, event-related potentials (ERPs), and EEG power were significantly affected by time on task. Brain stimulation did not significantly affect the evolution of subjective and objective executive vigilance performance, but significantly modulated spontaneous activity in the alpha and beta bands across the entire brain. The continuous enhancement of the prefrontal cortex increased P2 amplitude for the Oddball task, which was associated with the enhancement of the early stage of information processing. P3 amplitude had a temporary enhancement effect, which significantly decreased following a cognitive fatigue. tDCS had a continuous enhancement effect on N2 amplitude for the Go/Nogo task, which was associated with the enhanced inhibition of distracting stimuli. Together, the current data suggest that anodal tDCS over left DLPFC possibly enhances the early stage of relevant information processing and the inhibitory control of distracting stimuli during a continuous and monotonous vigilance task.

## Introduction

Many industrial, military, medical, and educational tasks require continuous vigilance for cognitive workload, including security personnel, driving, diagnostic medical screening, and industrial and air traffic control (Brookings et al., 1996; Gill, 1996; Näsholm et al., 2014; Korber et al., 2015; Reinerman-Jones et al., 2016). These long vigils can lead to vigilance decrement and a drop in cognitive efficiency, which results in increased reaction time, error rate, and even fatal consequences (Pattyn et al., 2008); therefore, it is necessary to investigate possible vigilance decrement countermeasures.

Vigilance is usually defined as the ability to sustain attention and remain alert to a particular stimulus over a prolonged period of time (Mackworth, 1948), which contains two different components of this function, referring to them as executive, and arousal vigilance (Luna et al., 2018, 2020). The executive vigilance mainly focused on the accuracy in the detection of an infrequent target and the inhibition of a frequent response, which was usually assessed by many behavioral tasks, such as the Sustained Attention to Response Task (SART) (Robertson et al., 1997), the Continuous Performance Test (Jacoby and Lavidor, 2018), or the Mackworth Clock Test (Lichstein et al., 2000). The arousal vigilance would be more involved in achieving and sustaining fast reactions to stimuli, without much control, i.e., without the consideration of alternative response options. The Psychomotor Vigilance Test (PVT) is a behavioral task developed to analyze the maintenance of arousal vigilance through time (Lim and Dinges, 2008). The executive vigilance decrement is generally found as a tendency to detect less critical events across time (Helton and Russell, 2015), due to a loss in the sensitivity to differentiate between unusual and usual events, or a change in the response bias (Thomson et al., 2016).

The dorsolateral prefrontal cortex (DLPFC) is an essential element in the neural network subserving executive functions like sustained attention (Toichi et al., 2004), inhibition of responses (Fassbender et al., 2004), and cognitive flexibility (Cools et al., 2004). Therefore, it is conceivable that the enhancement of activity in DLPFC may improve vigilance decrement and executive control in healthy subjects and patients with disorders, affecting the DLPFC function. Transcranial direct current stimulation (tDCS) has been established as a simple, effective, and safe method to modulate cortical excitability (Nitsche and Paulus, 2011) and cognitive functions (Wassermann and Grafman, 2005). In recent years, there has been a rapid expansion of research showing that tDCS is effective in enhancing healthy human vigilance performance (Mckinley et al., 2012; Al-Shargie et al., 2019). Mcintire et al. (2014, 2017, 2020) applied anodal tDCS to the pre-frontal cortex at 2 mA for 30 min to remediate the effects of sleep deprivation and to compare the behavioral effects of tDCS with those of caffeine and the results showed that tDCS prevented a decrement in arousal and executive vigilance and led to better subjective ratings for fatigue, drowsiness, energy, and composite mood compared to caffeine and control in sleep-deprived individuals. Over the left DLPFC, anodal tDCS successfully counteracted fatigability and reduced the fatigability-related increase in alpha power as well as the decline in both gating parameters during the 90 min executive vigilance task (Linnhoff et al., 2021). Furthermore, Nelson et al. (2014) found that tDCS over left DLPFC mitigated performance degradation in a 40 min simulated air traffic controller task, which required participants to detect infrequent critical signals. Anodal tDCS to the left DLPFC lead to a significant improvement in reaction time, an increase in P300 amplitude, and a decrease in N200 amplitude in the Flanker task (a conflict-related task) in a state-dependent manner: baseline ERP amplitudes conditioned the effects of tDCS (Dubreuil-Vall et al., 2019).

However, some studies showed diversity and inconsistent findings. For instance, 1.5 mA of tDCS (real vs. sham) for 20 min over the left DLPFC combined with cognitive fatigue induced by a 16 min TloadDback task (a sustained working memory paradigm), but tDCS was not effective to counteract the behavioral effects of cognitive fatigue or PVT performance (Borragán et al., 2018). Moreover, London and Slagter found that tDCS over left DLPFC did not affect attentional blink performance (Attentional blink reflects a limitation in processing resources required for the encoding and consolidation of target information in WM.) at the group level, and suggested that the pattern of results may be explained by an inverted U-shaped relationship between prefrontal excitability and attentional blink magnitude (London and Slagter, 2021). These results add to a growing body of work describing the null effects of tDCS on a variety of cognitive effects. In addition, Luna et al. (2020) examined the effects of online high-definition tDCS on the behavioral and electrophysiological functioning of attentional and vigilant components and found that online HD-tDCS effectively mitigated the executive vigilance decrement but not arousal vigilance. Another study also found that tracking performance at high attentional loads was significantly reduced in both cathodal and anodal stimulation conditions relative to sham, suggesting that tDCS may degrade attentional performance when cognitive networks become overtaxed and unable to compensate as a result (Roe et al., 2016).

From the above literature review, it is suggested that many factors could influence the effect of tDCS on sustained vigilance performance, such as online vs. offline stimulations, left vs. right DLPFC, high-definition vs. non-high-definition tDCS, high vs. low arousal state, task load, etc. Therefore, controlling for additional variables is important for the effects of tDCS. Although many studies showed that tDCS has a significant effect on executive vigilance (Mcintire et al., 2017; Dubreuil-Vall et al., 2019; Luna et al., 2020), whether this effect is due to enhanced processing of relevant visual inputs or enhanced inhibition of distracting stimuli and its neuroelectrophysiological basis remain to be proved. Therefore, in this study, we addressed the effects of tDCS on executive vigilance evolution across multi-sessions of monotonous stimuli, and the differences between sessions were compared. We hypothesize that the continuous vigilance task impairs subjective arousal state, behavioral performance, and neural activity. And anodal tDCS over left DLPFC would improve the behavioral performance and neural activity during the vigilance task.

## Materials and methods

### Participants

Participants were recruited from the Air Force Medical University. Inclusion criteria included: good general health, free of neurological diseases and psychiatric disorders, and no history of hearing and visual impairment. All participants underwent an informed consent process and all procedures were approved by the Air Force Medical University review board. Twenty-nine participants (all men; ages 20–25 years) were randomized to either active or sham tDCS from a pre-prepared randomization table. There were no significant group differences (real vs. sham) in any demographic or personal characteristics (see Table 1). During the experiment, participants were not allowed to consume any form of alcohol, caffeine, or nicotine products. At the conclusion of the study, participants were thanked for their time and compensated with 500 RMB.

**TABLE 1:** Participant demographic and personality characteristics (Mean ± *SD*).

Personal characteristics	Sham (*n* = 14)	Real (*n* = 15)	*t*	*p*
Age (years)	21.02 ± 1.27	21.29 ± 1.41	–0.52	0.605
Weight (kg)	70.50 ± 8.05	71.73 ± 7.01	–0.44	0.663
Height (m)	1.78 ± 0.04	1.76 ± 0.04	1.61	0.119
BMI (kg/m^2^)	22.15 ± 2.18	23.17 ± 1.56	–1.45	0.159
Extraversion	28.43 ± 6.07	27.47 ± 3.89	0.50	0.619
Agreeableness	35.50 ± 6.02	36.80 ± 4.99	–0.63	0.531
Conscientiousness	30.93 ± 5.64	33.93 ± 5.20	–1.49	0.147
Neuroticism	20.79 ± 6.09	22.20 ± 4.02	–0.74	0.464
Openness	35.21 ± 5.35	36.40 ± 4.66	–0.64	0.529
EI	5.65 ± 0.71	5.51 ± 1.10	0.40	0.692
SSS	2.43 ± 1.02	2.27 ± 0.70	0.50	0.620
TMD	80.14 ± 11.45	84.47 ± 14.12	–0.89	0.381

BMI, body mass index; EI, emotional intelligence; SSS, the Stanford sleepiness scale score; TMD, the total mood disturbance.

G*Power 3.1.9.4 (Faul et al., 2007) was used to determine whether the sample size in this study was enough for the experimental design (i.e., a within-between interaction). *A priori* analysis demonstrated that considering a medium effect size *f* = 0.25, power of 1-β = 0.8, α = 0.05 and number of groups = 2. When the number of measurements = 4, the estimated sample size was 24; When the number of measurements = 5, the estimated sample size was 22. Therefore, the sample size N = 29 adopted in this study is sufficient. Furthermore, *post hoc* analysis showed that given an effect size of *f* = 0.25, α = 0.05, total sample size = 29, 1-β = 0.80, number of groups = 2, number of measurements = 4, corr among rep measures = 0.5, sample size was enough to observe a within-between interaction with a power of 1-β = 0.89 (power of 1-β = 0.93, when number of measurements = 5). In addition, according to a previous study, effect size *f* = 0.176 (Luna et al., 2020), compromise analysis showed that the sample size was enough to observe a within-between interaction with a power = 0.812 (i.e., *f* = 0.176, total sample size = 29, 1-β = 0.80, number of groups = 2, number of measurements = 4, corr among rep measures = 0.5; power of 1-β = 0.834 when number of measurements = 5), which met the acceptable 80%.

### Vigilance task

Mackworth Clock Test (Vigilance Task): The Vienna Test System (VTS-version 23, Schuhfried ^®^, Austria) was used to assess vigilance performance. The vigilance task was an adopted version of the Mackworth clock test with parameters adopted from Mcintire et al. (2017) and run on a standard desktop computer. The participant was shown a visual display with a white dot moving against a black back ground. The illuminated dot jumps in a clockwise direction *via* a step-wise progression along a circular path. The participant’s task is to respond by pressing a button as fast as possible on the keyboard with his preferred index finger when a “double jump” occurs, which is considered a target signal. The target stimuli are presented occasionally and pseudo-randomly. The dot moves in 2-s steps. The whole task lasts for approximately 33 min in all. 32 critical stimuli (double jumps) occur during this period. The response was defined as a correct hit when it occurred less than 1 s after the target signal appeared and a false alarm if the reaction occurred outside this time range (0.1–1.0 s). Undetected targets were defined as misses. The vigilance performance is reflected by the accuracy of the response to the target stimulus.

### Oddball task

The oddball task was utilized to reflect participants’ cognitive fatigue by allowing us to investigate vigilance decrement when cognitive fatigue was present (Clayton et al., 2015). In an oddball paradigm, visual stimuli were presented in a continuous stream, and participants must detect the presence of an oddball stimulus. In this study, there were two kinds of stimuli. One stimulus “X” with a high probability (about 80%) is called standard stimuli, and another stimulus “O” with a small probability (about 20%) is called deviant stimuli. The signal duration was 100 ms, with an interval of 1,000 ms. Reaction operation to target stimuli may interfere with event-related potential (ERP) components, for instance, P300 (Nakata et al., 2004; Smith et al., 2006). In this study, participants were required to count the number of the deviant stimuli “O” without pressing any button and then reported to the experimenter that number when the task finished. Participants conducted this task 5 times in this study, in which the number of deviant stimuli was random from 54 to 66, and the standard stimulus was 4 times the deviant stimulus.

### Go/Nogo task

The task was a standard Go/Nogo paradigm, where participants were presented with a stream of stimuli composed of any of two different white letters (“S” or “H”). Stimuli were presented at random with equal probability against a black background, each appearing eighty times [to avoid the effect of different stimulus frequency on the results (Luck, 2014)]. Participants were instructed to press the space bar of a keyboard as fast as possible in response to the letter “S” (“go”). No response was required for the letter “H” (“no-go”). The signal duration was 1,000 ms, with an interval of 1,000 ms. This task took about 5 min. The Go/Nogo paradigm is often used in attentional studies and is a standard accepted measure of inhibition control (Plewnia et al., 2013; Miller et al., 2015). The inhibitory control ability is reflected by the inhibition of non-target stimulus, that is, the less the false alarms, the better the inhibitory control ability, and vice versa.

### Subjective questionnaires

The Stanford sleepiness scale (SSS): SSS is a frequently used arousal state self-report scale (Zhang et al., 2021). This scale has a single item with a scale ranging from one (“Feeling active and vital; alert; wide awake.”) to seven (“Almost in reverie; sleep onset soon; lost struggle to remain awake.”).

The rating scale mental effort (RSME): To measure participants’ subjective mental effort in each vigilance task, we used RSME (Zijlstra, 1993). This scale has good validity to measure mental effort and has been used in many studies (van der Linden et al., 2003; Herlambang et al., 2019). RSME uses a vertical scale from 0 to 150 with some anchors from absolutely no effort to extreme effort.

The big five factor traits: The big five-factor personality traits were measured using the Chinese version of the 44-item Big Five Personality Inventory (Zhai et al., 2013). This scale contains 44 items to measure the Big Five factors: eight items for extraversion, eight items for neuroticism, nine items for conscientiousness, nine items for agreeableness, and ten items for openness to experience. The response is a five-point Likert-type scale ranging from 1 (strongly disagree) to 5 (strongly agree).

Wong and Law emotional intelligence scale (WLEIS): Emotional intelligence (EI) was assessed by a Chinese version of the self-report WLEIS (Wong and Law, 2002), which consists of 16 brief items. The scale includes four subscales: self-emotion appraisals (SEA), others’ emotion appraisals (OEA), use of emotion (UOE), and regulation of emotion (ROE). The response format of the WLEIS is a 7-point scale (1 = strongly disagree to 7 = strongly agree). For consistency of interpretation, higher scores on all dimensions indicate higher levels of EI.

Abbreviated Profile of Mood States (POMS): The Abbreviated POMS is a questionnaire to measure mood disturbances for adults (Grove and Prapavessis, 1992). This questionnaire consists of 40 items with seven subscales, namely tension–anxiety, depression–dejection, anger–hostility, fatigue–inertia, vigor–activity, confusion–bewilderment, and esteem–related affect. These items are scored by using a Likert scale from 0 (not at all) to 4 (extremely). The Total Mood Disturbance (TMD) was calculated by adding all the negative subscales and subtracting the positive subscale. Then, the score was added with a constant 100 to eliminate the negative value of TMD. A higher score indicates a higher mood disturbance.

### Transcranial direct cerebral stimulation

Anodal tDCS was targeted to the left DLPFC using a bipolar montage with the anode placed at F3 and the cathode placed over the contralateral supraorbital area at Fp2 (according to the 10–20 electrode placement system) (Gaynor et al., 2020). This is a common montage in studies targeting left DLPFC for enhancing cognitive function (Nienow et al., 2016). TDCS leads were connected to 25 cm^2^ saline-soaked sponges on the scalp. Active stimulation group was delivered for 30 min at 1.5 mA (current density = 0.06 mA/cm^2^), including 30 s of ramp-up/-down time. Sham tDCS consisted of a 15 s ramp up followed by a 15 s ramp down of the current. The device used was a NE STARSTIM tCS^®^ (Barcelona, Spain). Adverse effects were systematically measured after each tDCS group using a questionnaire similar to the one proposed by Brunoni et al. (2011). Participants responded as “absent,” “mild,” “moderate,” or “severe” to each of the items. Examples of items included were “Headache,” “Tingling,” “Itching,” “Sleepiness,” and “Acute mood change.”

### Procedure

**FIGURE 1:**
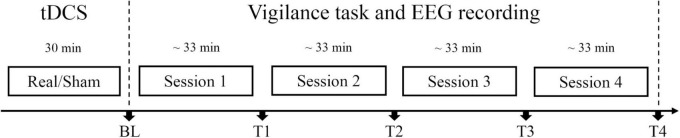
Experiment protocol. Participants in real tDCS group or sham tDCS group received real tDCS or sham tDCS for 30 min, respectively. Each session lasted for about 33 min. At each time point [BL (baseline), T1, T2, T3, and T4], subjective and objective vigilance parameters were assessed: subjective arousal level *via* the Stanford Sleepiness Scale, performance, and event-related potentials (ERPs) in Oddball and Go/Nogo tasks. Electroencephalography (EEG) data were collected throughout the vigilance task.

Before the procedure was carried out, the institutional review board on human research approved this study, and informed consent was obtained from all volunteers. In this study, participants were randomly assigned to the anodal tDCS group or sham tDCS group. When arrived at the laboratory, participants rested to be calm for about 5 min and then received anodal tDCS or sham tDCS for 30 min. After brain stimulation, participants filled out the adverse effect questionnaire and then completed one session of the SSS, one session of the Oddball task and one session of the Go/Nogo task at baseline. The priorities of these two tasks were counterbalanced for participants in each group to eliminate possible effects of the task order. Then, the participants repeated the vigilance task for four sessions, each for about 33 min. The SSS, Oddball task, and Go/Nogo task were completed at time points T1∼T4, respectively (see Figure 1). Electroencephalography (EEG) data were collected throughout the vigilance task. The whole experiment procedure lasted about 5 h.

### Electroencephalography recording and preprocessing

A 64-channel Neuroscan SynAmps2 amplifier recorded brain electrical activity with a sampling frequency of 1,000 Hz (Neuroscan Inc., United States). EEG was recorded continuously by Ag/AgCl-electrodes mounted in an elastic cap, with those electrode sites according to the 10–20 electrode placement system. Scalp recordings were referenced online to the electrode between Cz and CPz and re-referenced to the average of the left and right mastoids through offline analysis. The vertical electrooculogram (VEOG) data were recorded from electrodes above and below the left eye. The horizontal electrooculogram (HEOG) data were monitored by placing electrodes at the outer canthi of both eyes. EEG data were collected with all electrode impedances kept below 10 kΩ. Offline data analyses were conducted using the EEGLAB toolbox of MATLAB R2013b. The continuous EEG signals were filtered using a band-pass filter from 0.5 to 40 Hz with a notch filter at 50 Hz. Filtered data were segmented into epochs of –200 to 800 ms after the stimulus and baseline-corrected relative to an interval of –200 to 0 ms for ERP analyses. Eye movement artifacts were removed using independent component analysis (ICA). Trials contaminated with large artifacts (peak to peak deflection exceeding 80 μV) were excluded.

### Data analysis

After baseline correction, averages for the standard and deviant stimuli were computed separately for each time point (BL, T1, T2, T3, and T4) and stimulation group (real, sham). For ERPs analysis, difference waves were computed by subtracting average ERPs of the standard stimuli from ERPs of the deviant stimuli. We determined time windows centered on the peak by visually inspecting individual data and measured local peak amplitude as defined by Luck (2014). In line with previous studies, P2 was detected as occurring at about 160–260 ms from the onset of the stimulus, N2 detects in the time window of about 220–320 ms, and P3 in the time window of about 300–500 ms (Chen et al., 2021). ERPs Analysis was confined to the electrodes at anterior frontal Fz, central Cz, and posterior parietal Pz. A peak detection analysis was performed on single-subject difference waves measured using the local maximum (P2 and P3) or minimum (N2) during the time windows after stimulus onset. For EEG analysis, two participants were excluded due to EEG signal quality (one from the real tDCS group and one from the sham group). The FFT algorithm was applied to the decontaminated data to obtain the absolute power spectral density (PSD) from 1 to 40 Hz after EEG artifacts such as eye movement and muscle were removed. To analyze the changes in EEG activity over time, the average PSD value was calculated for each session of the vigilance task (about 33 min). EEG power spectrum within delta (0.1–3.5 Hz), theta (3.5–8 Hz), alpha (8–12 Hz), and beta (12–24 Hz) bands for electrode F3, F4, and the entire brain were analyzed.

Behavioral data included response time, accuracy, and false alarm of Oddball and Go/Nogo tasks recorded by E-Prime 3.0 software. Based on the signal detection theory, sensitivity (d’) and response bias (β) were calculated using the method provided by Macmillan and Kaplan (1985). Data were statistically analyzed using IBM SPSS software 22. We used an independent-samples *t*-test to compare participants’ demographic or personal characteristics between the anodal and sham groups. The repeated-measures analyses of variance (rm-ANOVA) were conducted. Two-way rm-ANOVAs were used to analyze the effects of time (BL, T1, T2, T3, T4, or Session 1, Session 2, Session 3, Session 4), group (real and sham) and their interaction on subjective drowsiness, the response time (only for correct responses), accuracy rates, false alarm, d’ and log β. For the ERP component, the 5 (time point: BL, T1, T2, T3, T4) × 2 (group: real, sham) × 3 (electrode site: Fz, Cz, Pz) rm-ANOVAs were conducted to analyze the effects on amplitude and latency of each ERP component. All *post hoc* paired comparisons of the group based on the Sidak correction.

## Results

In order to exclude the effects of participants’ characteristic variables on experimental results, we used independent-samples *t*-test to compare participants’ age, weight, height, BMI, personality traits, emotional intelligence, arousal state, and the TMD between real and sham groups. As shown in Table 1, there were no significant differences between sham and real tDCS groups on any demographic or personality characteristics.

### Behavioral results for vigilance task

**FIGURE 2:**
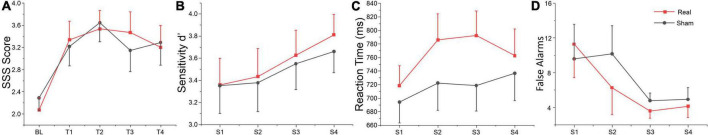
Behavioral data. S1, S2, S3, and S4 indicate session 1 to session 4, respectively. **(A)** Self-reported vigilance for the SSS at each time point. **(B)** Sensitivity d’, **(C)** reaction time, and **(D)** false alarms during the vigilance task across each session. The error bars indicate the standard error of the mean (SEM), the same below.

The average score in drowsiness (SSS score) over time-on-task separately for real and sham tDCS groups are shown in Table 2. The repeated measures ANOVA revealed a significant main effect for time-on-task. Sidak paired comparisons showed SSS score at BL was significantly lower than which at any other time point (T1, T2, T3, and T4) (see Figure 2A). Neither main effect was found for group nor interaction between group and time. Participants’ subjective vigilance was significantly affected by time on the vigilance task.

**TABLE 2:** Behavioral results for vigilance task.

Variables	Session			Group			Session × Group		
			
	*F*	*p*	η^2^	*F*	*p*	η^2^	*F*	*p*	η^2^
Target hits	2.49 (3.81)	0.066	0.08	0.38 (1.27)	0.543	0.01	0.12 (3.81)	0.949	0.004
Reaction time	2.44 (3.81)	0.071	0.08	1.07 (1.27)	0.31	0.04	0.72 (3.81)	0.545	0.03
False alarms	4.1 (3.81)	0.034*	0.13	0.15 (1.27)	0.702	0.006	0.59 (3.81)	0.513	0.02
Sensitivity d’	3.80 (3.81)	0.013*	0.12	0.06 (1.27)	0.803	0.002	0.11 (3.81)	0.951	0.004
Log β	1.95 (3.81)	0.128	0.07	1.26 (1.27)	0.272	0.05	0.83 (3.81)	0.481	0.03
Mental effort	2.73 (3.81)	0.074	0.09	0.21 (1.27)	0.651	0.01	1.55 (3.81)	0.221	0.05
SSS score	11.0 (4.108)	<0.001***	0.29	0.0002 (1.27)	0.988	<0.001	0.43 (4.108)	0.726	0.02

***p < 0.001 and *p < 0.05. Note, the Greenhouse–Geisser sphericity correction was applied when necessary, the same below.

**FIGURE 3:**
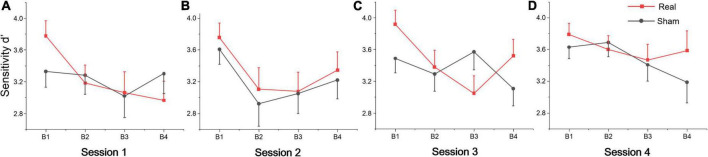
Sensitivity d’ per block across four sessions: **(A)** Session 1, **(B)** Session 2, **(C)** Session 3, and **(D)** Session 4. Each session was divided into four blocks (B1, B2, B3, and B4).

The repeated measures of ANOVA also revealed that there were significant main effects of session for false alarms, sensitivity d’ and mental effort, and also marginally significant session effects for target hits and reaction time, as shown in Table 2. No significant group or interaction effects were found for any index. Sensitivity d’ gradually increased, while the false alarms gradually decreased, indicating that subjects’ executive vigilance gradually improved both for two groups with time on task (see Figures 2B–D). To detect possible changes in performance at each session of the vigilance task, we divided each session into four blocks, with 8 target stimuli (double jump) per block. The ANOVA revealed that there were significant main effects of block for sensitivity d’ at session 1 [*F*(3, 81) = 3.74, *p* = 0.014, η^2^ = 0.12], session 2 [*F*(3, 81) = 8.99, *p* < 0.001, η^2^ = 0.25] and session 3 [*F*(3, 81) = 3.25, *p* = 0.029, η^2^ = 0.1]. Meanwhile, a significant interaction effect was found on session 3 [*F*(3, 81) = 4.24, *p* = 0.008, η^2^ = 0.14], where d’ in the real tDCS group decreased significantly at block 3 relative to block 1 (*p* = 0.002) (see Figure 3). All *post hoc* comparisons were based on Sidak correction. On the whole, there were no significant differences between real and sham groups for vigilance performance. Participants’ executive vigilance gradually improved for the two groups across four sessions, decreasing and then increasing at each session.

### Electroencephalography frequency analysis

**FIGURE 4:**
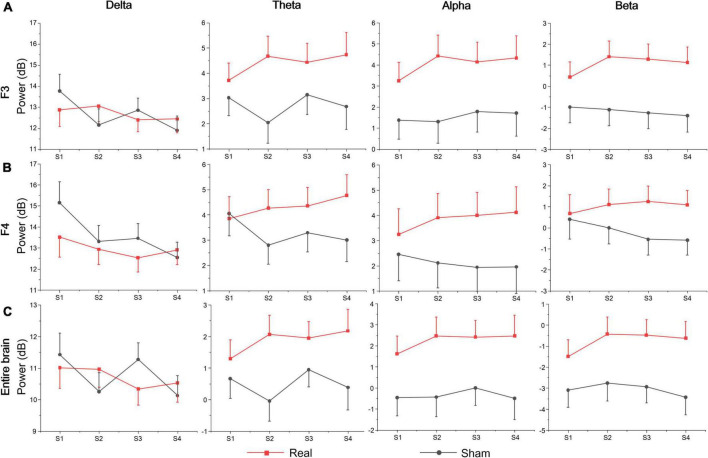
EEG band power of **(A)** F3, **(B)** F4, and **(C)** the entire brain average at each session.

Table 3 shows the statistics of the ANOVAs performed on the EEG power spectrum within delta (0.1–3.5 Hz), theta (3.5–8 Hz), alpha (8–12 Hz), and beta (12–24 Hz) bands for electrode F3, F4, and the entire brain average. There was a significant group effect for the beta band at electrode F3, where the beta power of the real tDCS group was significantly greater than which of the sham group, and alpha power also had a marginally significant group effect (see Figure 4). For electrode F4, there was a significant session effect for the delta band, with a gradual decrease across different sessions. But no significant group or interaction effects were found for any EEG band power at F4. Furthermore, the group effects of alpha and beta power were significant for the entire brain. Therefore, compared with the sham group, real tDCS significantly increased the electrical activity in the left dorsolateral prefrontal and the entire brain cortex, particularly high-frequency electrical activity, i.e., alpha and beta.

**TABLE 3:** Results of analysis of variance (ANOVA) on normalized values of EEG power within the delta, theta, alpha, and beta bands at electrodes F3, F4, and the entire brain.

EEG power		Session			Group			Session × Group		
				
		*F*	*p*	η^2^	*F*	*p*	η^2^	*F*	*p*	η^2^
F3	Delta	2.35 (3.75)	0.079	0.09	0.001 (1.25)	0.98	<0.001	1.85 (3.75)	0.145	0.07
	Theta	0.62 (3.75)	0.603	0.02	2.72 (1.25)	0.112	0.1	2.29 (3.75)	0.085	0.08
	Alpha	1.34 (3.75)	0.27	0.05	3.62 (1.25)	0.069	0.13	0.84 (3.75)	0.475	0.03
	Beta	0.45 (3.75)	0.721	0.02	5.72 (1.25)	0.025*	0.19	1.01 (3.75)	0.393	0.04
F4	Delta	4.85 (3.75)	0.012*	0.16	0.46 (1.25)	0.505	0.02	1.71 (3.75)	0.172	0.6
	Theta	0.42 (3.75)	0.667	0.02	0.99 (1.25)	0.329	0.04	2.33 (3.75)	0.105	0.09
	Alpha	0.08 (3.75)	0.923	0.003	1.68 (1.25)	0.206	0.06	1.1 (3.75)	0.339	0.04
	Beta	0.22 (3.75)	0.884	0.01	1.61 (1.25)	0.217	0.06	1.23 (3.75)	0.299	0.05
Entire brain	Delta	1.73 (3.75)	0.169	0.07	0.01 (1.25)	0.931	0.001	1.76 (3.75)	0.163	0.07
	Theta	0.75 (3.75)	0.523	0.03	3.41 (1.25)	0.077	0.12	1.77 (3.75)	0.161	0.07
	Alpha	0.94 (3.75)	0.427	0.04	4.77 (1.25)	0.039*	0.16	0.59 (3.75)	0.625	0.02
	Beta	0.96 (3.75)	0.415	0.04	5.53 (1.25)	0.027*	0.18	0.62 (3.75)	0.608	0.02

*p < 0.05.

According to the above analysis, at each session of the vigilance task, the sensitivity to target stimuli decreased first and then increased, but the sensitivity increased gradually across the full four sessions for both real and sham tDCS groups. It indicates that in session 1, the participants’ were already at a relatively low state of executive vigilance, and increased vigilance was tDCS unrelated to the brain stimulation. Based on core cognitive functions of sustained vigilance, the results of the Oddball task (infrequent target stimuli to detect the process of relevant visual inputs) and the Go/Nogo task (equal frequency for target and non-target stimuli to detect inhibition of distracting stimuli) are analyzed below.

### Oddball task results

**FIGURE 5:**
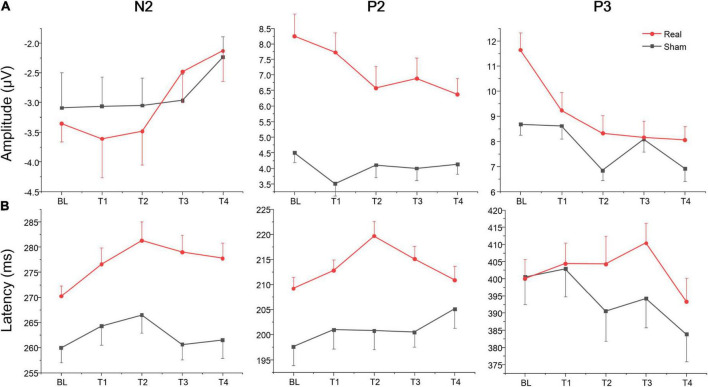
Amplitude and latency of ERPs component for Oddball task at each time point. **(A)** Mean amplitudes of N2, P2, and P3. **(B)** Mean latencies of N2, P2, and P3.

The rate (reported number/targets number; the closer the mean was to 1, the higher the accuracy was) and difference value (the value of reported number minus targets number; the closer the mean was to zero, the higher the accuracy was) of Oddball task for the real and sham group at each time point (BL, T1, T2, T3, and T4). The repeated measure ANOVA showed no significant main effect or interaction both for the rate and difference value at each time point (BL, T1, T2, T3, and T4) or group (real and sham) (see Supplementary material). The average amplitudes and latencies of N2, P2, and P3 for groups (real and sham) and brain regions (Fz, Cz, and Pz) across sessions (BL, T1, T2, T3, and T4) were compared *via* three-factor repeated measure ANOVA (see Figure 5). As shown in Table 4, there were significant time effects for N2, P2, and P3. The ANOVA also revealed significant group, region, and interaction effects between time and group for P2 and P3, but not for N2. Sidak paired comparisons showed N2 amplitude at T4 was significantly greater than which at BL (*p* = 0.094), T1 (*p* = 0.002), and T2 (*p* = 0.003), respectively. Contrasts showed that P2 amplitudes in the anodal group at each time point were all greater than in the sham group (all *p*s < 0.05). And P3 amplitude in the real group only at BL was greater than sham group (*p* = 0.025). For the effects of the brain region, contrasts revealed that P2 amplitude at P_Z_ was significantly lower than F_Z_ (*p* < 0.001) and C_Z_ (*p* = 0.017), and also revealed that P3 amplitude at F_Z_ was significantly lower than C_Z_ (*p* = 0.001) and P_Z_ (*p* < 0.001) (see Supplementary material). No significant effects on the brain region were found for the N2 component.

**TABLE 4:** Amplitude and latency of ERPs component for Oddball task.

Effect	N2			P2			P3		
			
	*F*	*p*	η^2^	*F*	*p*	η^2^	*F*	*p*	η^2^
**Amplitude**									
Time	4.82 (4.72)	0.002**	0.21	5.06 (4.72)	0.001**	0.22	13.5 (4.72)	<0.001***	0.43
Group	0.05 (1.75)	0.829	0.001	25.34 (1.75)	<0.001***	0.25	4.59 (1.75)	0.035*	0.06
Region	1.05 (2.75)	0.357	0.03	8.82 (2.75)	<0.001***	0.19	15.51 (2.75)	<0.001***	0.29
Group × Region	0.42 (2.75)	0.659	0.01	0.88 (2.75)	0.418	0.02	0.69 (2.75)	0.506	0.02
Time × Group	1.07 (4.72)	0.38	0.06	4.12 (4.72)	0.005**	0.19	4.43 (4.72)	0.003**	0.2
Time × Region	0.53 (8.146)	0.829	0.03	0.5 (8.146)	0.854	0.03	0.55 (8.146)	0.819	0.03
Time × Group × Region	0.37 (8.146)	0.933	0.02	0.14 (8.146)	0.997	0.01	0.4 (8.146)	0.922	0.02
**Latency**									
Time	3.84 (4.72)	0.007	0.18	1.98 (4.72)	0.107	0.1	1.53 (4.72)	0.202	0.78
Group	24.05 (1.75)	<0.001***	0.24	16.6 (1.75)	<0.001***	0.18	1.35 (1.75)	0.249	0.02
Region	23.84 (2.75)	<0.001***	0.39	4.83 (2.75)	0.011*	0.11	0.13 (2.75)	0.875	0.004
Group × Region	0.29 (2.75)	0.748	0.01	2.33 (2.75)	0.104	0.06	0.26 (2.75)	0.771	0.01
Time × Group	1.22 (4.72)	0.312	0.06	2.18 (4.72)	0.08	0.11	1.0 (4.72)	0.413	0.05
Time × Region	0.31 (8.146)	0.96	0.02	0.39 (8.146)	0.923	0.02	0.23 (8.146)	0.984	0.01
Time × Group × Region	1.55 (8.146)	0.145	0.08	0.76 (8.146)	0.635	0.04	0.39 (8.146)	0.927	0.02

***p < 0.001; **p < 0.01; and *p < 0.05.

A separate ANOVA revealed a significant time effect for N2 latency, which showed that latency at BL was shorter than at T2 (*p* = 0.003). The ANOVA also revealed significant group and region effects for N2 and P2, respectively. These contrary results revealed that N2 and P2 latencies in the real group were both greater than in the sham group (both *p*s < 0.001). For brain regions, N2 latency at P_Z_ was shorter than F_Z_ (*p* < 0.001) and C_Z_ (*p* = 0.003), respectively, and latency at C_Z_ was shorter than F_Z_ (*p* = 0.002). Contrasts also revealed that P2 latency at P_Z_ was both shorter than F_Z_ (*p* = 0.017) and C_Z_ (*p* = 0.066) (see Supplementary material). No other significant results were found for this metric. Waveforms at each electrode are provided in Supplementary material.

### Go/Nogo task results

#### Behavioral results

**FIGURE 6:**
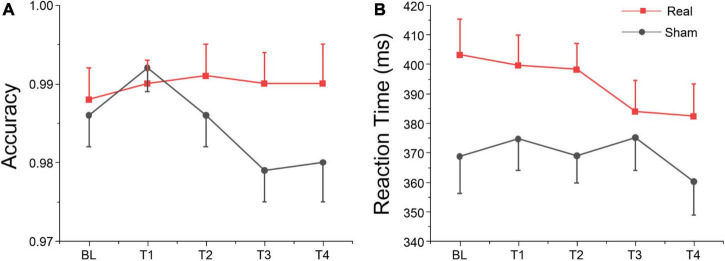
Go/Nogo task results at each time point. **(A)** Accuracy for no-go trials. **(B)** Reaction time for go trials.

We analyzed the accuracy of the no-go trials of the Go/Nogo test (i.e., the proportion of no-go trials in which participants withheld their response), and the reaction time (RT) on go trials of the Go/Nogo test. The ANOVA only revealed a marginally significant group effect for RT on go trials, but no significant session or interaction effects were found for both variables as shown in Table 5. According to Figure 6, simple effect analysis showed that accuracy on no-go trials in real group at T3 was greater than sham group [*F*(1, 27) = 4.91, *p* = 0.035], and reaction time at T2 was greater than sham group [*F*(1, 27) = 5.32, *p* = 0.029]. These results suggest that tDCS over left DLPFC might enhance the participants’ inhibitory control for non-target stimuli, although no significant main, or interaction was found.

**TABLE 5:** Behavioral results for Go/Nogo task.

Variables	Session			Group			Session × Group		
			
	*F*	*p*	η^2^	*F*	*p*	η^2^	*F*	*p*	η^2^
Accuracy on no-go trials	1.2 (4.108)	0.316	0.04	1.91 (1.27)	0.179	0.07	1.24 (4.108)	0.298	0.04
Reaction time	2.23 (4.108)	0.115	0.08	3.25 (1.27)	0.083	0.11	1.26 (4.108)	0.293	0.04

#### Event-related potentials results

**FIGURE 7:**
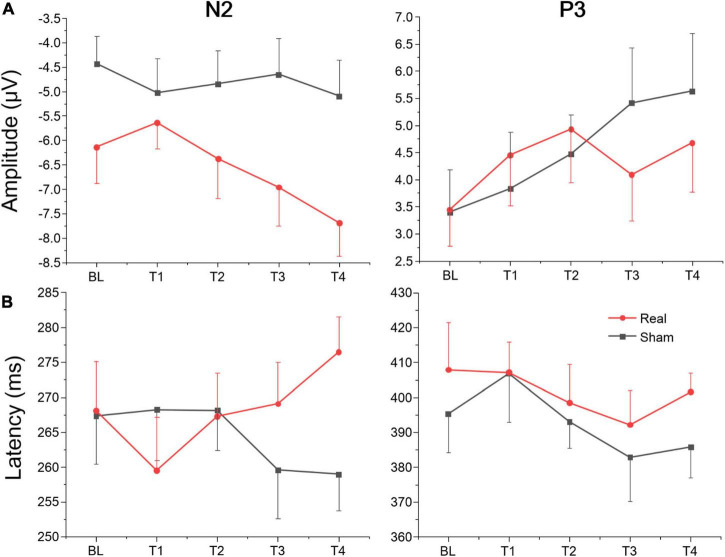
Amplitude and latency of ERPs component for Go/Nogo task at each time point. **(A)** Mean amplitudes of N2 and P3. **(B)** Mean latencies of N2 and P3.

The average amplitudes and latencies of N2 and P3 for groups (real and sham) and brain regions (F_Z_, C_Z_, and P_Z_) across sessions (BL, T1, T2, T3, and T4) were compared *via* three-factor repeated measure ANOVA (see Figure 7). As shown in Table 6, there were significant time, region, and interaction effects between time and group both for N2 and P3. Sidak paired comparisons showed that N2 amplitude at T1 was greater than at T4 (*p* = 0.049) for the anodal group. And N2 amplitudes in anodal group at BL (*p* = 0.085), T3 (*p* = 0.046), and T4 (*p* = 0.015) were lower than sham group, respectively. Contrasts also showed that showed P3 amplitude at BL was significantly lower than which at T2 (*p* = 0.037), T3 (*p* = 0.038), and T4 (*p* = 0.001), respectively. P3 amplitude slightly decreased at T3 for the anodal group, while the sham group gradually increased. For the effects of the brain region, contrasts revealed that N2 amplitude at F_Z_ was significantly lower than P_Z_ (*p* = 0.037) and also revealed that P3 amplitude at F_Z_ was significantly greater than C_Z_ (*p* = 0.016) and P_Z_ (*p* < 0.001), and amplitude at C_Z_ was greater than P_Z_ (*p* = 0.002) (see Supplementary material).

**TABLE 6:** Amplitude and latency of ERPs component for Go/Nogo task.

Effect	N2			P3		
		
	*F*	*p*	η^2^	*F*	*p*	η^2^
**Amplitude**						
Time	3.97 (4.72)	0.006**	0.18	4.47 (4.72)	0.003**	0.2
Group	12.71 (1.75)	0.001**	0.15	0.11 (1.75)	0.745	0.001
Region	3.39 (2.75)	0.039*	0.08	20.49 (2.75)	<0.001***	0.35
Group × Region	0.11 (2.75)	0.897	0.003	0.07 (2.75)	0.93	0.002
Time × Group	2.57 (4.72)	0.045*	0.13	2.98 (4.72)	0.025*	0.14
Time × Region	0.18 (8.146)	0.994	0.01	0.51 (8.146)	0.848	0.03
Time × Group × Region	0.22 (8.146)	0.987	0.01	0.44 (8.146)	0.895	0.02
**Latency**						
Time	0.8 (4.72)	0.532	0.04	3.5 (4.72)	0.011*	0.16
Group	0.58 (1.75)	0.45	0.01	1.41 (1.75)	0.239	0.02
Region	3.06 (2.75)	0.053	0.08	13.49 (2.75)	<0.001***	0.27
Group × Region	0.34 (2.75)	0.712	0.01	0.02 (2.75)	0.982	<0.001
Time × Group	5.03 (4.72)	0.001**	0.22	0.54 (4.72)	0.709	0.03
Time × Region	0.54 (8.146)	0.823	0.03	0.89 (8.146)	0.523	0.05
Time × Group × Region	0.43 (8.146)	0.903	0.02	0.75 (8.146)	0.645	0.04

***p < 0.001; **p < 0.01; and *p < 0.05.

A separate ANOVA revealed a significant interaction between time and group for N2 latency, which showed latency at T1 was shorter than at T4 (*p* = 0.094) for the anodal group, and at T4 latency, in the anodal group, was greater than in the sham group (*p* = 0.025). The ANOVA also revealed a significant time effect for P3. Sidak paired comparisons revealed that P3 latency at T1 was significantly greater than which at T3 (*p* = 0.005). For brain regions, N2 latency at P_Z_ was greater than F_Z_ (*p* = 0.031) and C_Z_ (*p* = 0.047), respectively; similarly, P3 latency at P_Z_ was also greater than F_Z_ (*p* < 0.001) and C_Z_ (*p* < 0.001) (see Supplementary material). No other significant results were found for this metric. Waveforms at each electrode are provided in Supplementary material.

## Discussion

Sustained attention would mainly rely on monitoring of current attentional focus, enhanced processing of relevant visual inputs, and inhibition of distracting stimuli (Clayton et al., 2015). This study examined the effects of tDCS over the left DLPFC on sustained attention to a series of monotonous stimuli over a prolonged period of time, which reflect energization of task-relevant processes and inhibition of task-irrelevant processes, respectively. Specifically, we measured the effects of tDCS on the development of subjective vigilance, behavioral performance, and neural activity for the Oddball task and Go/Nogo task. The results suggested that tDCS had different effects on the metrics of vigilance which were negatively influenced by vigilance decrement.

With the duration of the experiment, participants’ subjective arousal level presented an inverted U-shaped change trend and reached the peak at session 2. We found similar results for vigilance task performance, in which sensitivity d’ was the lowest in session 1 and then gradually improved both for the real and sham groups. A possible explanation for this effect is due to the familiarity or learning effect of multi-session vigilance tasks (Jacoby and Lavidor, 2018). Alternatively, according to resource theory, continuous vigilance task interruptions, including rest breaks or other task interruptions, would both result in vigilance performance improvement, which is especially true when the interrupting task actually overlaps much with the specific processing resources of the primary vigilance task (Helton and Russell, 2015). This result is consistent with that of Axelrod et al. (2015) (montage: anode electrode at left F3 electrode site, cathode electrode over the contralateral supraorbital ridge). To further explore the neuroelectrophysiological basis of the above results, we analyzed the EEG power spectrum during each vigilance session within the delta, theta, alpha, and beta bands at electrodes F3, F4, and the entire brain average. Compared with the sham group, real tDCS significantly increased electrical activity in the left dorsolateral prefrontal and the entire brain cortex, particularly high-frequency electrical activity, i.e., alpha and beta. Similar results were also obtained in previous studies, for example, tDCS to left DLPFC improved alpha power of the prefrontal cortex in patients with a minimally conscious state (Cavinato et al., 2019), and beta PSD of left DLPFC (El-Hagrassy et al., 2021). Although it remains unclear for many oscillatory frequencies whether their activity reflects the engagement or disengagement of sustained attention (Kamiński et al., 2012; Clayton et al., 2015), some studies still reflected positive results of oscillatory EEG features and cognitive function. For example, alpha power reflected improved attention when averaged across the scalp (Makeig and Jung, 1995). And the anodal stimulation provoked an increase in cognitive performance, which highly correlated with an increase in beta activity after the stimulation (Ashikhmin et al., 2017). Therefore, the behavioral and neural electrical activity results of sustained vigilance task diverged over time-on-task. This result is in line with the previous report that one single session of anodal tDCS over the left DLPFC did not improve subjective exhaustion but prevented fatigability-related increases in occipital alpha power (Linnhoff et al., 2021).

ERPs are related to cognitive functions such as recognition, judgment, memory, and decision, reflecting different aspects of the cognitive process, and are the “window” to understand the cognitive function of the brain (Luck, 2014). We analyzed neurocognitive state of sustained vigilance task over time *via* the Oddball task paradigm, which is often used to study cognitive fatigue or alertness to infrequent stimuli (Clayton et al., 2015). P3 is a positive wave observed about 300 ms after the appearance of the deviant stimulus, and the amplitude was the highest near the P_Z_ point. P3 amplitude was positively correlated with the number of mental resources invested (Duncan-Johnson and Donchin, 1977), and it was an endogenous component mainly related to psychological factors (Kutas et al., 1977). In this study, we found that P3 amplitude was significantly affected by time-on-task and showed a gradually decreasing trend. In addition, the P3 amplitude of the real tDCS group was significantly higher than that of the sham group at baseline. However, this difference disappeared at T1, suggesting that tDCS could increase P3 amplitude before the vigilance task, and the number of mental resources invested in the real group was higher than that in the sham group. After one session vigilance task, this mental resource decreased significantly in the real group. According to Mcintire et al., 2014, 2017, tDCS to the left DLPFC significantly increased vigilance for up to 6 h, while the effects in our study only lasted about 1.5 h. This suggested that tDCS over left DLPFC may temporarily increase the number of mental resources, however, this effect is significantly reduced after acute cognitive fatigue induced by a series of monotonous stimuli over a prolonged period of time, which does not require too much mental resource and habituation (Luck, 2014). Compared with P3, there are relatively few studies on P2. Generally, P2 is an exogenous ERP component, which is affected by the physical characteristics of the stimulus (Luck, 2014). It is distributed around the centro-frontal and the parieto-occipital areas of the scalp (Lenartowicz et al., 2010) and has been considered to reflect the early stage of information processing (Ciecko-Michalska et al., 2012) and is related to effective information selection, attention allocation, and memory recognition (Lenartowicz et al., 2010). From the mean P2 amplitude perspective, the difference in amplitude between trial types implies a change of attention in stimulus switching (Luck, 2014; Stefanics et al., 2014). The current study revealed that P2 amplitude in the real tDCS group was significantly higher than in the sham group, even though a slight downward trend across sessions. This result is in agreement with a previous study which found that tDCS to the left DLPFC led to a significant increase in the time patients withhold their response in Go-trials waiting for the Stop Signal to appear, which was correlated with a significant increase in P200 amplitude (Dubreuil Vall et al., 2020). There is a wide range and diversity of factors that have been found to affect the characteristics of the P2, but its amplitude is generally associated with selective attention to visual stimuli (Phillips and Takeda, 2009). Therefore, we could interpret the increase in P2 amplitude as a modulation in selective attention when searching for infrequent target stimuli. According to the above analysis, although tDCS over left DLPFC significantly increased P2 amplitude in the Oddball task, there was no significant difference in vigilance performance between real and sham tDCS groups. This result is in line with the development trend of high-frequency band (i.e., alpha and beta) power during the vigilance task. This may be related to participants’ basal state. A study showed that transcranial alternating current stimulation (tACS) at alpha frequency improved executive vigilance in the SART only when arousal was low (Martínez-Pérez et al., 2022). In this study, both groups were at a good arousal level and emotional state before tDCS (see Table 1). Another possible explanation is that the difficulty of the vigilance task may affect results. The Mackworth Clock Test adopted in this study is a simple and monotonous sustained attention task which not consume too many mental resources (Mackworth, 1948). Therefore, the amplitude of endogenous component P3, which governs the allocation of mental resources, decreased gradually without impairing external task performance with task-on-time.

In addition to the processing of relevant visual inputs, executive vigilance performance may also be influenced by the inhibition of distracting stimuli (Clayton et al., 2015). The Go/Nogo paradigm is a standard accepted measure of inhibition control (Plewnia et al., 2013; Miller et al., 2015). Participants were instructed to press the space bar of a keyboard as fast as possible in response to the non-target stimuli, which appeared with the same probability as target stimuli (no response) to avoid the effect of different stimulus frequencies on the results (Luck, 2014). Unlike other cognitive tasks, in which fast responses are associated with better performance, the opposite is true in the Go/Nogo test. Better performance is achieved when participants slow down their responses to non-target stimuli on go trials to successfully refrain from responding to the target stimuli on no-go trials (Allan et al., 2009; Smilek et al., 2010). We analyzed the accuracy of the no-go trials of the Go/Nogo test (i.e., the proportion of no-go trials in which participants withheld their response), and the reaction time (RT) on go trials of the Go/Nogo test. It is noteworthy that, no significant main or interaction between time-on-task and group was found, which may be due to the relatively small sample size. But simple effect analysis showed that accuracy on no-go trials in the real group at T3 was greater than in the sham group, and reaction time at T2 was greater than the sham group. These results suggest that tDCS over left DLPFC might enhance the participants’ inhibitory control for non-target stimuli. These results were in agreement with previous studies in which transcranial electrical stimulation to the left DLPFC improved executive function or mitigated the executive vigilance decrement (Dubreuil-Vall et al., 2019; Luna et al., 2020). The brain mechanism of response inhibition measured by the Go/Nogo task was analyzed in this study. Under the Go/Nogo experimental paradigm, many researchers have found ERP components of inhibition control related to the prefrontal lobe. The two most important ERP components are (1) N2 about 200–300 ms after stimuli appear, which has a larger negative amplitude under a no-go trial than in a go trial. (2) P3 about 300–600 ms after stimuli, which has a larger positive amplitude under a no-go trial than a go trial. Both components were considered to be closely related to response inhibition (Bekker et al., 2004; Smith et al., 2006, 2008). In our study, we also found a larger negative amplitude for N2 and a larger positive amplitude for P3 at prefrontal site Fz. Results disclosed a different evolution of the N2 component in sham and real groups across the four vigilance task sessions. N2 amplitude remained stable in the sham group but increased gradually from T1 to T4 in the real group. N2 latency slightly decreased in the sham group, while increasing gradually from T1 to T4 in the real group. However, there was no significant difference in the P3 component between both groups. Many studies have debated whether the N2 and P3 effects of the no-go trial occurred in response inhibition, and the focus of the debate was mainly no-go P3 effect, which may be affected by reaction operations (Salisbury et al., 2004; Bekker et al., 2005; Dimoska et al., 2006). Some studies have found that a no-go P3 effect in the prefrontal cortex only appeared in experimental conditions requiring keystroke response, but not in counting conditions (Nakata et al., 2004; Smith et al., 2006). In this study, N2 amplitude in the real tDCS group increased gradually, while the sham group stayed at a relatively low level. Furthermore, there was no significant difference in P3 amplitude evolution between both groups, due to the possible action effect. From the behavioral results, no significant main or interaction between time-on-task and group was observed, but simple effect analysis showed that the real group had better inhibitory control performance, which is related to a continuous increase in N2 amplitude, where real tDCS significantly changed the amplitude and latency of N2, thus possibly improving inhibitory control performance. This result is in line with a previous report of anodal tDCS over the left DLPFC inducing a larger N2 amplitude for executing the Flanker Task (a well-established experimental paradigm to assess the executive function) (Dubreuil-Vall et al., 2019).

In summary, anodal tDCS over left DLPFC relative to sham tDCS did not significantly improve the evolution of a prolonged period of executive vigilance task in this study, but significantly changed neural activity in the brain cortex, i.e., alpha and beta power and as well as ERP components for Oddball and Go/Nogo tasks. Several reasons may explain this discrepancy. First, because Oddball and Go/Nogo tasks and subjective questionnaires were conducted between each session of vigilance tasks, the interruptions and repetition between tasks may be helpful to the improvement of vigilance (Helton and Russell, 2015; Jacoby and Lavidor, 2018). Second, tDCS was conducted in the offline mode before cognitive fatigue occurred. Both groups were at a good arousal level and emotional state before tDCS, resulting in no significant enhancement of tDCS in a state-dependent manner (Martínez-Pérez et al., 2022). Third, the vigilance task adopted in this study is a simple and monotonous sustained attention task which not consume too many mental resources. Thus, even increased brain excitability would not significantly change behavioral performance. Task paradigms that consume more cognitive resources, such as *N*-back, continuous computing task, Stroop test, etc. need to be further verified. Last but not least, perhaps due to the relatively small sample size, there are no significant differences in some behavioral results for real and sham tDCS groups, which may be one reason for the inconsistency between neural activity and behavioral results. The present study possesses several limitations of note. First, to simplify EEG data collection, all the recruited participants were men, which may have affected the generalizability of our results. Therefore, further investigation is required to evaluate the applicability of these findings in women. Second, due to the relatively small sample size, no significant differences were observed in some behavioral results, especially for the Go/Nogo task. Larger sample size is needed to confirm the results. Third, we did not obtain eye movement, ECG, or electrodermal activity to more accurately analyze neuropsychological changes during the vigilance task. Future studies should emphasize the dynamic neuropsychological effect of tDCS on vigilance.

In summary, the present study demonstrated the effects of anodal tDCS over the left DLPFC on executive vigilance evolution during a continuous monotonous condition. Subjective sleepiness, task performance, and electrical activity in the cerebral cortex were significantly affected by task-on-time. Brain stimulation did not significantly affect the evolution of subjective and objective vigilance performances, but significantly modulated spontaneous activity in the alpha and beta bands across the entire brain, with higher activation levels in the left prefrontal lobe than in the right. From the perspective of cognitive neural function, the current results suggest that anodal tDCS over left DLPFC possibly enhances the early stage of relevant information processing and the inhibitory control of distracting stimuli during a continuous and monotonous vigilance task.

## Data availability statement

The original contributions presented in this study are included in the article/Supplementary material, further inquiries can be directed to the corresponding author/s.

## Ethics statement

The studies involving human participants were reviewed and approved by Air Force Medical University. The patients/participants provided their written informed consent to participate in this study.

## Author contributions

JD and WH conceived and designed the experiments. JD and HW contributed substantially to the acquisition, analysis, and interpretation of the data and drafted the article. LY and CW were responsible for the resources and software. SC and TZ participated in data collection and analysis. JM, ZW, and XC contributed to review the literature and interpret the results. All authors contributed to the article and approved the submitted version.
