# Supplementary material for: The neuroelectrophysiological and behavioral effects of transcranial direct current stimulation on executive vigilance under a continuous monotonous condition
Source: Front Neurosci. 2022 Sep 7;16:910457. doi: 10.3389/fnins.2022.910457 (PMC9489920; doi:10.3389/fnins.2022.910457)
Supplement: Supplementary file 1 [file Table_1.DOCX]

Supplementary Materials

# 1. Results of Oddball task

FIGURE 1 showed the rate (reported number / targets number) and difference value (the value of reported number minus targets number) of Oddball task for real and sham group at each time point (BL, T1, T2, T3 and T4). The repeated measure ANOVA showed no significant main effect or interaction both for the rate and difference value at each time point (BL, T1, T2, T3 and T4) or group (real and sham).


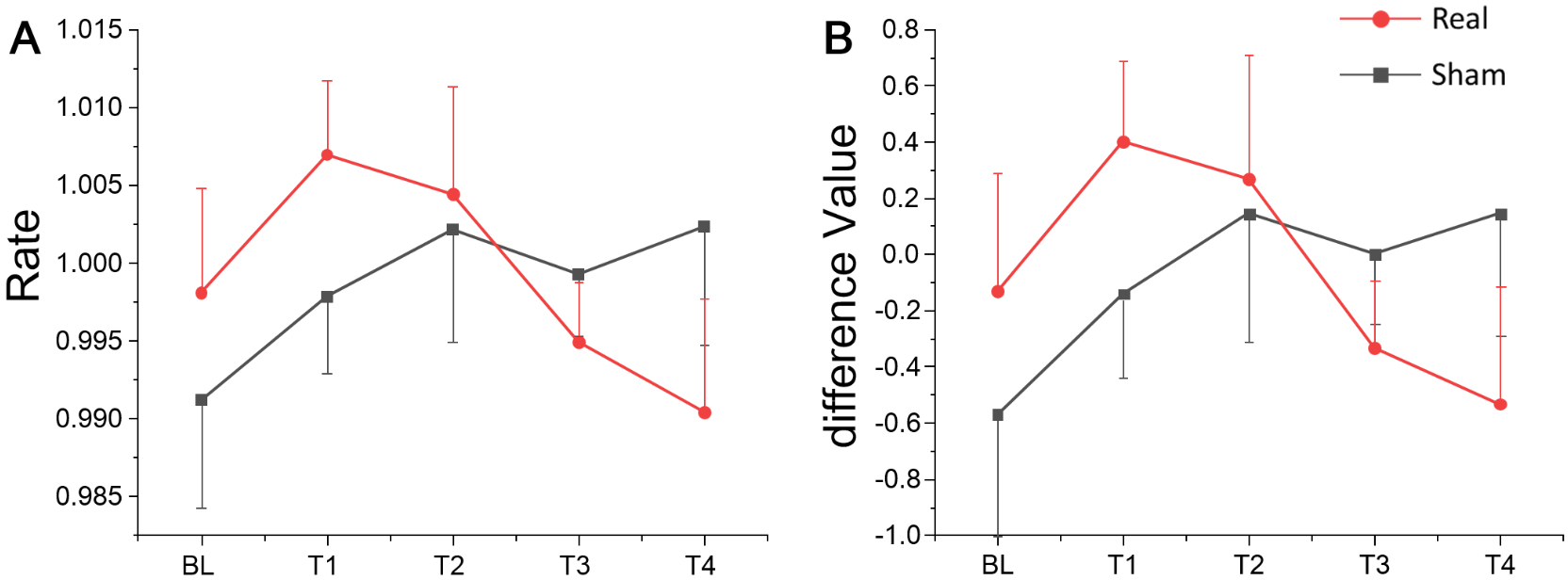


**FIGURE 1 |** (A) The rate (reported number / targets number) and (B) difference value (the value of reported number minus targets number) of Oddball task for real and sham group at each time point (BL, T1, T2, T3 and T4).

FIGURE 2 showed the waveform of Oddball task for real and sham group at each time point (BL, T1, T2, T3 and T4) respectively.


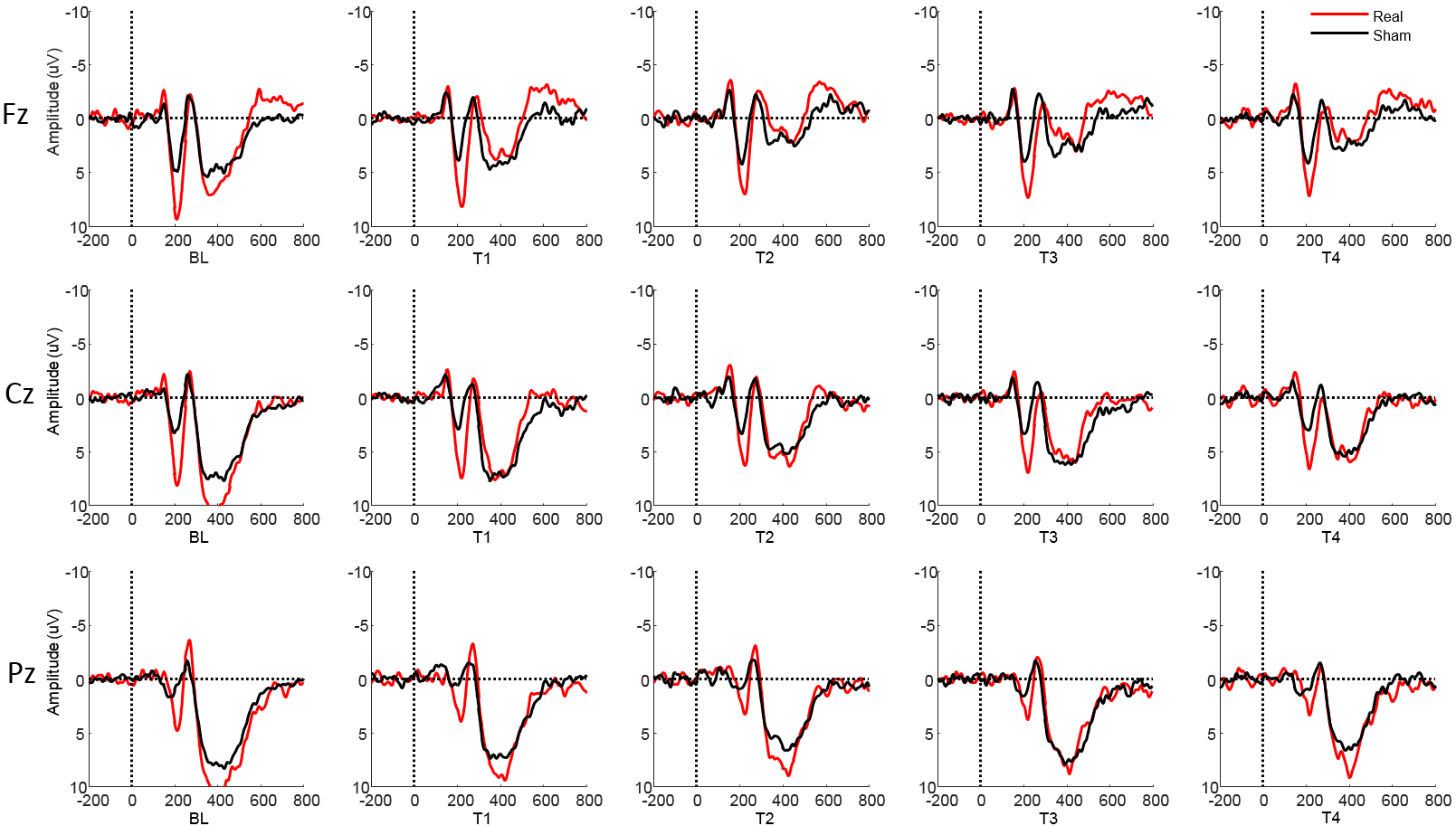


**FIGURE 2 |** The waveform of Oddball task for real and sham group at each time point (BL, T1, T2, T3 and T4).

For the effects tDCS on different brain regions, as shown in FIGURE 3,the repeated measure ANOVA revealed that P2 amplitude at P_Z_ was significantly lower than F_Z_ (*p* < 0.001) and C_Z_ (*p* = 0.017), and also revealed that P3 amplitude at F_Z_ was significantly lower than C_Z_ (*p* = 0.001) and P_Z_ (*p* < 0.001). Furthermore, N2 latency at P_Z_ was shorter than F_Z_ (*p* < 0.001) and C_Z_ (*p* = 0.003) respectively, and latency at C_Z_ was shorter than F_Z_ (*p* = 0.002). Contrasts also revealed that P2 latency at P_Z_ was both shorter than F_Z_ (*p* = 0.017) and C_Z_ (*p* = 0.066). No interaction effect was found between region and time.

**A N2 P2 P3**


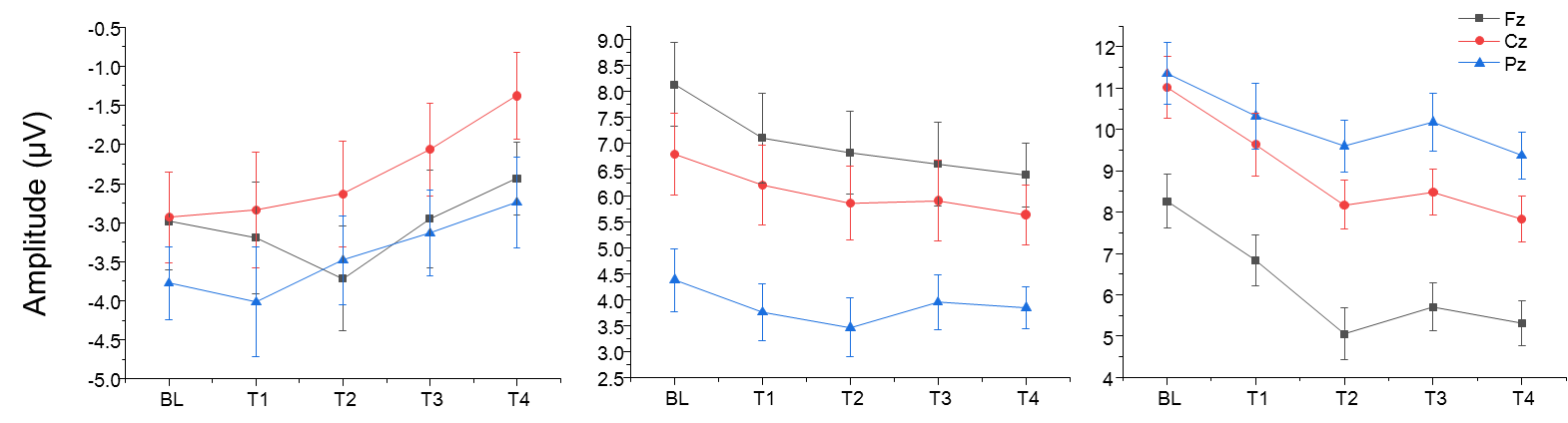


**B**


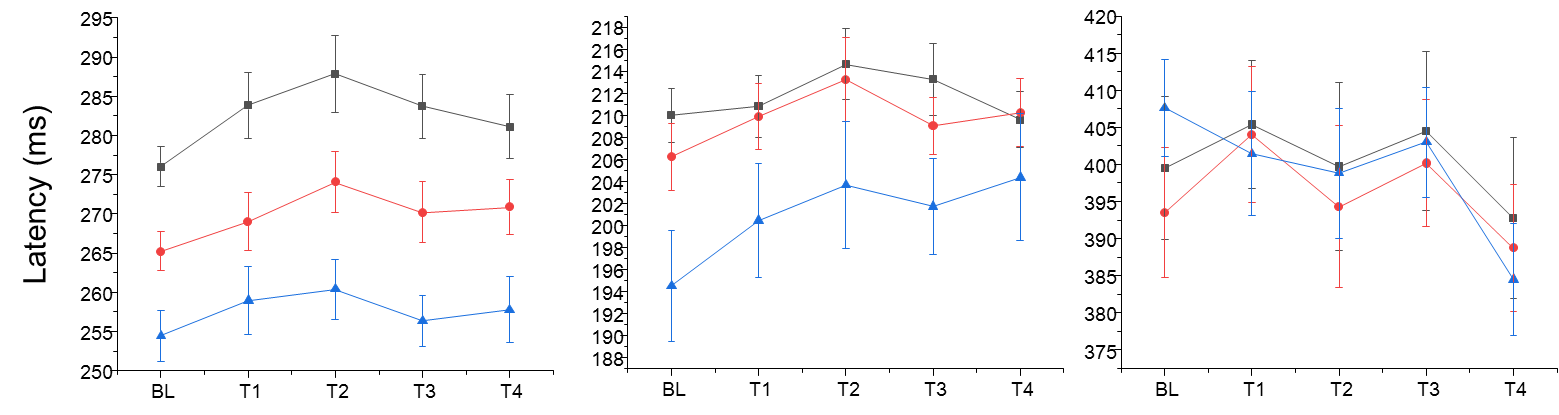


**FIGURE 3 |** Amplitude and latency at electrode F_Z_, C_Z_ and P_Z_ of ERPs component for Oddball task at each time point. (A) Mean amplitudes of N2, P2 and P3. (B) Mean latencies of N2, P2 and P3.

# 2. ERP results of Go/Nogo task

FIGURE 4 showed the waveform of Go/Nogo task for anodal and sham group at each time point (BL, T1, T2, T3 and T4) respectively.


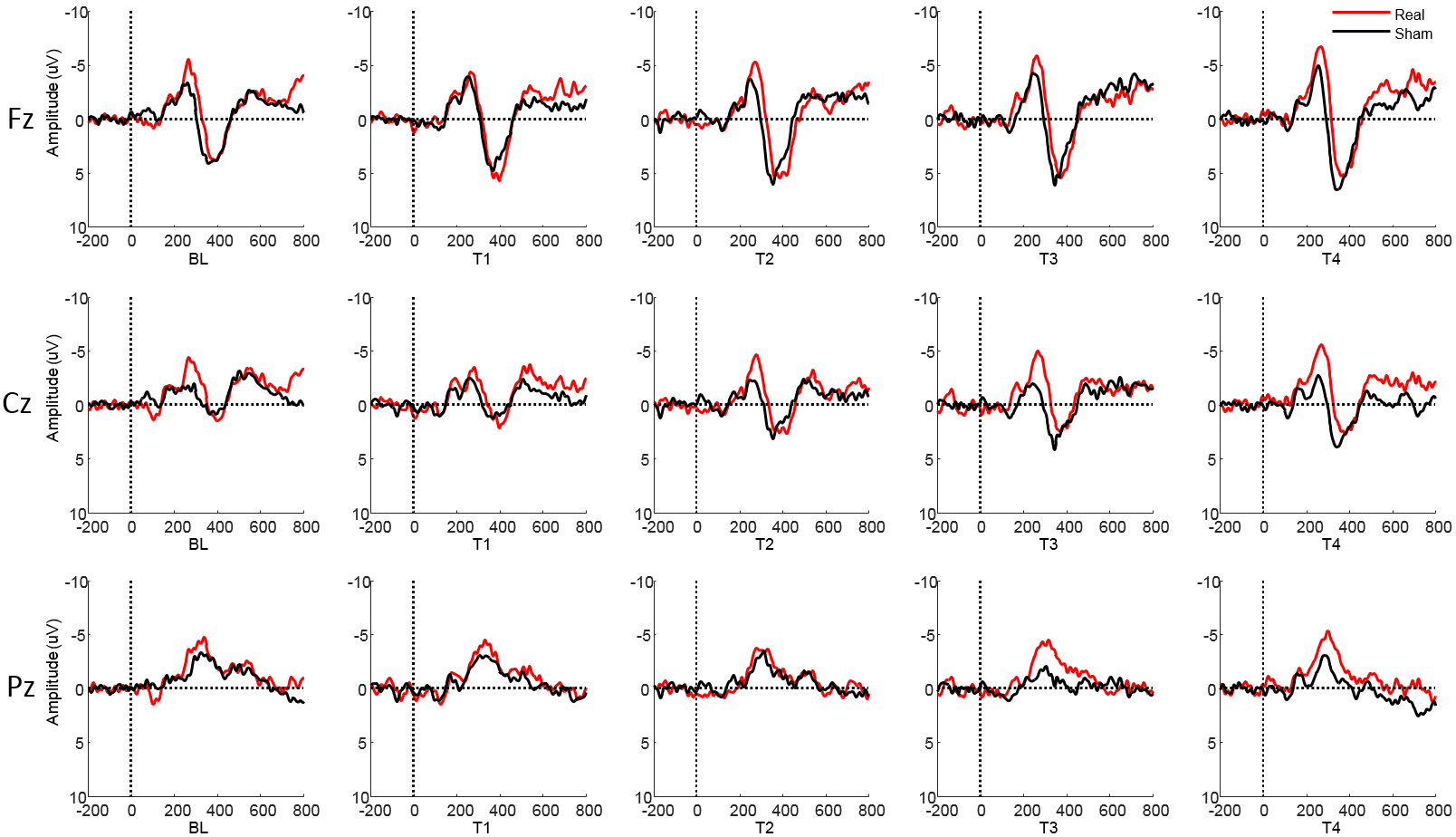
**FIGURE 4 |** The waveform of Go/Nogo task for anodal and sham group at each time point (BL, T1, T2, T3 and T4).

For the effects tDCS on different brain regions, as shown in FIGURE 5, the repeated measure ANOVA revealed that N2 amplitude at F_Z_ was significantly lower than P_Z_ (*p* = 0.037) and also revealed that P3 amplitude at F_Z_ was significantly greater than C_Z_ (*p* = 0.016) and P_Z_ (*p* < 0.001), and amplitude at C_Z_ was greater than P_Z_ (*p* = 0.002). Furthermore, N2 latency at P_Z_ was greater than F_Z_ (p = 0.031) and C_Z_ (*p* = 0.047) respectively; similarly, P3 latency at P_Z_ was also greater than F_Z_ (*p* < 0.001) and C_Z_ (*p* < 0.001). No interaction effect was found between region and time.

**A N2 P3**


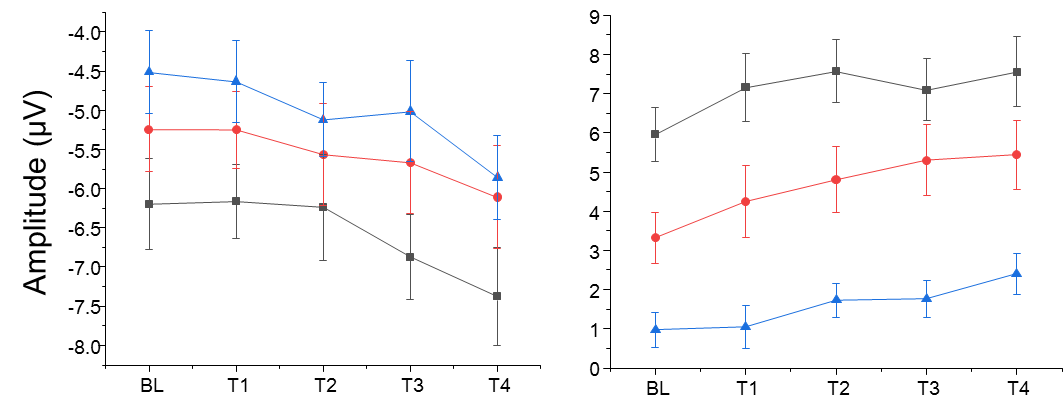


**B**


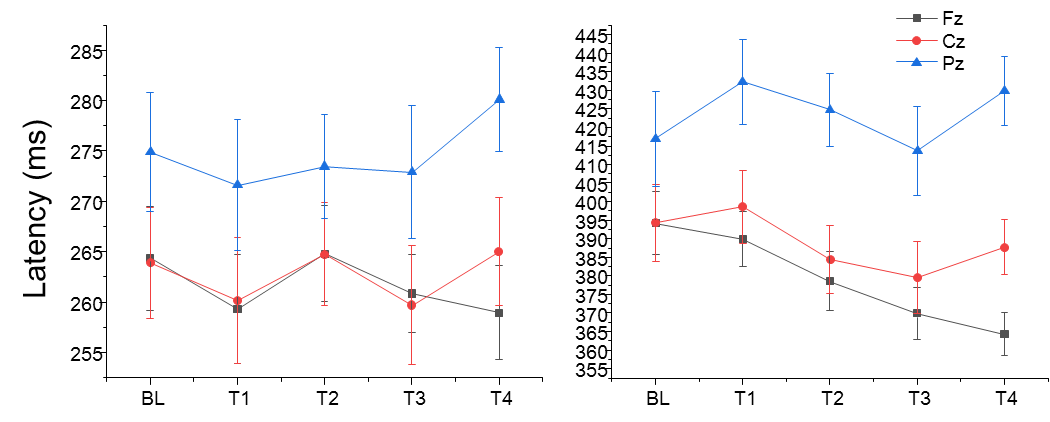


**FIGURE 5 |** Amplitude and latency at electrode F_Z_, C_Z_ and P_Z_ of ERPs component for Go/Nogo task at each time point. (A) Mean amplitudes of N2 and P3. (B) Mean latencies of N2 and P3.
